# The application of ^1^H NMR to explore the taste difference caused by taste‐active metabolites of different Chinese sauce‐stewed beef

**DOI:** 10.1002/fsn3.1773

**Published:** 2020-07-21

**Authors:** Yi Yang, Ying Wang, Daodong Pan, Yuyu Zhang, Jun He, Qiang Xia, Jinxuan Cao

**Affiliations:** ^1^ State Key Laboratory for Managing Biotic and Chemical Threats to the Quality and Safety of Agro‐products Ningbo University Ningbo China; ^2^ Key Laboratory of Animal Protein Food Processing Technology of Zhejiang Province Ningbo University Ningbo China; ^3^ Beijing Advanced Innovation Center for Food Nutrition and Human Health Beijing Technology and Business University Beijing China

**Keywords:** Chinese sauce‐stewed beef, multivariate data analysis, NMR, sensory evaluation, taste‐active metabolites

## Abstract

In this study, we developed a method for the quantification of taste‐active metabolites of Chinese commercial sauce‐stewed beef by using ^1^H NMR spectroscopy coupled with multivariate data analysis. This method was applied to analyze the taste difference which caused by taste‐active metabolites of different Chinese sauce‐stewed beef. Beef samples demonstrated to consist of 25 metabolites, including amino acids, sugars, organic acids, nucleic aides, and their derivatives. PC1 and PC2 explained a total of 85.1 and 13.1% of variables, respectively. Metabolites such as isoleucine, histidine, glutamate, pyroglutamate, sucrose, lactate, creatine, carnitine, and creatinine were kept at a higher levels compared with other metabolites in the four products. Sensory evaluation was also done to help analyze the taste difference. This microcosmic approach of using high‐resolution NMR spectrometry to analyze beef products has rarely been reported. This work established a feasible method to distinguish the taste difference of different Chinese sauce‐stewed beef.

## Graphical abstract

The proportions and combinations of metabolites explained the specific taste of Chinese sauce‐stewed beef. NMR provides detailed information on specific components of complex mixtures. Taste is the major factor which decided by biochemical composition.

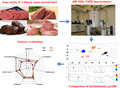

## 1 INTRODUCTION

Nuclear magnetic resonance (NMR) spectroscopy, one of the most common investigative techniques, has been proven to be an extremely useful analytical technique for metabolomics research. NMR‐based metabolomic analysis has potential applications to analyze food components, assess food quality, and monitor food consumption and diet metabolomics profiling (Chen, Zhao, Wu, He, & Yang, 2020; Wishart, 2008). It has been applied to differentiate the geographical origin of beef samples (Jung et al., 2010), identify molecular markers in dried beef, and assess the effect of irradiation on ground beef (Shintu, Caldarelli, & Franke, 2007; Zanardi et al., 2015), tested the effect of vacuum impregnated fish gelatin and grape seed extract of tilapia fillets during storage (Zhao, Wu, Chen, & Yang, 2019). Chen, Ye, Chen, Zhan, and Lou (2017) also monitored the change of metabolites regarding molecular nutritional characteristic of vinasse pike eel during pickling processing. NMR has several advantages of unknown metabolites detection, quick determination, high accuracy, nondestruction and require of small samples (Bertram, Oksbjerg, & Young, 2010). Compared with high performance liquid chromatography (HPLC), NMR could provide structural and quantitative information, and is extremely useful in the meat products (Brennan, 2014). Not limited to animals, NMR is also applied in the detected metabolites of bacteria and plant (Chen et al., 2019; Liu et al., 2018; Zhao, Chen, Wu, He, & Yang, 2020). Up to now, NMR has been widely applied in detecting metabolites of different fields but rarely in sauce‐stewed beef product.

Chinese sauce‐stewed beef (CSSB), a Chinese ethnic meat product, is popular in the whole area of China. It is processed by boiling raw bovine leg meat with spices (cinnamon, star anise, clove, amomum kravanh, and cortex cinnamomi) and seasonings (salt, sugar, white wine, and soy sauce), then stewing in old brine at 75°C for about 2 hr. In the history for more than hundred years, there were 4 typical CSSB (Wanlong, Hengsheng, Pingyao, and Yueshengzhai) in Chinese market. Wanlong and Hengsheng are popular in southern area of China, especially in Jiangsu, Anhui, and Zhejiang provinces, while Pingyao and Yueshengzhai are popular in northern area, especially in Beijing, Shanxi, and Qinghai provinces. North flavor was formatted around Beijing area with the character of “fresh and crisp,” while south flavor was formatted around Jiangsu province with mellow and agreeable sweet taste. In various regions of China, the processing methods of CSSB were different, since people have dissimilar preferences on the flavor and taste (Zeng et al., 2016).

Taste is one of the most important aspects which contributed to the sensory quality of meat products (Aaslyng & Meinert, 2017). The main taste‐active compounds of stewed beef juice have been considered as free amino acids, sugars, organic acids, and nucleotide degradation products (Schlichtherle‐Cerny & Grosch, 1998). Among of them, amino acids present diverse characteristics. Alanine and glycine contribute to the sweet flavor in meat, whereas valine, tyrosine, isoleucine, leucine, and phenylalanine produce bitter flavor (Pereira‐Lima, Ordoñez, de Fernando, & Cambero, 2000). Kim, Kemp, and Samuelsson (2016) reported that histidine, asparagine, succinate, and lactate provided sour taste in meat. Metabolites were the origin of various flavors, which lead to taste different in meat products. In order to distinguish the taste of 4 styles of CSSB (Wanlong, Hengsheng, Pingyao, and Yueshengzhai), ^1^H NMR‐based metabolomics analytical method of taste‐active metabolites variation in different CSSB was established.

Therefore, the aims of this study were (a) to detect the main taste‐active metabolites of CSSB products, (b) to explore the taste difference caused by taste‐active metabolites of different CSSB products, and (c) to evaluate the ability of ^1^H NMR to characterize the taste‐active metabolites in beef products.

## 2 MATERIALS AND METHODS

### 2.1 Materials and sampling

CSSB included Wanlong (Wanlong meat product CO., Ltd.), Hengsheng (Anhui hengsheng industrial Co., Ltd.), Pingyao (Pingyao Beef Group Co., Ltd.), and Yueshengzhai (Beijing Yanqi Yueshengzhai Halal Food Co., Ltd.) were obtained from local factories before vacuum packaging. Wanlong and Hengsheng belong to southern products. Pingyao and Yueshengzhai belong to northern products. Ten pieces of Wanlong, Hengsheng, Pingyao, and Yueshengzhai (with an average weight of approximate 1–1.2 kg) were obtained and marked as A, B, C, and D group in sequence. Each piece of CSSB was trimmed into uniform size along the muscle lines. All samples were labeled and packaged with tin foil, respectively, then prepared at −40°C before extraction procedure.

### 2.2 Extraction procedure

The method of extraction is referred to a previous literature (Yang et al., 2017). The frozen beef samples were thawed and cut into small cubes (about 0.4 × 0.4 × 0.4 cm) for convenient mincing. 100 g of small cubes of each piece was minced and mixed by a mini mincing machine (model JYL‐C012, Jiuyang Co., Ltd.). Among the 100 g samples, 400 mg samples was extracted with 1,000 µl methanol/water (2:1, v/v) via homogenization for 10 times at 12,400 *g* (i.e., 30 s homogenization followed by a 30 s break) and centrifuged for 10 min at 12,400 × *g* and 4°C. The above experimental steps repeated twice. After combining the resultant supernatants, methanol and small amount of water were removed in vacuum; the supernatants were lyophilized into powder. Each sample was added into 550 µl Na^+^/K^+^ buffer solution (0.1 M, 50% D_2_O, 0.001% TSP, containing 0.1% NaN_3_ and 0.001% sodium 3‐trimethylsilyl [2, 2, 3, 3‐d_4_] propionate (TSP) prepared with 50% D_2_O, pH 7.39), and vortex‐mixed. The mixture centrifuged at 12,400 × *g* and 4°C for 10 min. After centrifugation, 500 µl supernatant of each extract was transferred into a 5 mm outer diameter NMR tube for NMR analysis.

### 2.3 NMR analysis

All the ^1^H NMR spectra of extracts were executed at 298 K on a Bruker Avance 600 MHz Spectrometer (Bruker Biospin) combined with an inverse detection probe under the operating condition of 600.13 MHz for ^1^H. To collect the spectra of metabolite profiles of each sample, a standard one‐dimensional pulse sequence (RD‐90°‐t_1_‐90°‐t_m_‐90°‐acquisition) was conducted with a mixing time (t_m_, 80 ms) and a weak irradiation to suppress the water signal during recycle delay (RD, 2 s). A 90° pulse length was set to 13.8 µs; the parameter t_1_ was set to 4 µs. A total of 32 transients were collected into 32 k data points with a spectral width of 20 ppm. All free induction decays (FIDs) employed an exponential window function with a 1 Hz line broadening factor prior to Fourier transformation (FT).

### 2.4 Data analysis

With the completion of phase and baseline corrections, ^1^H NMR spectra (δ 9.0–0.2) were integrated into regions with equal width of 0.004 ppm (2.4 Hz). The spectral regions containing methanol signals (δ 3.38–3.354) and residual water (δ 4.9–4.7) were eliminated. Each bucketed region was normalized to the total sum of spectral integral to compensate for the whole concentration difference. After that, the normalized NMR data sets were analyzed by multivariate data analysis (the software package SIMCA‐P^+^, version 11.0, Umetrics). Principal component analysis (PCA) was executed using mean‐centered scaling; the scores and loading plots expressed final consequence. To acquire a general view of variation among groups, each point in scores represented an individual sample, yet the loading plots represented magnitude and manners of NMR signals to classification. The orthogonal projection to latent structure with discriminant analysis (OPLS‐DA) method with sevenfold cross‐validation, and unit‐variance scaling was performed to further analyze any intrinsic biochemical dissimilarities between the different styles of CSSB. The whole OPLS‐DA models were validated by cross‐validated residuals (CV‐ANOVA) approach with *p < *.05 as significant level. The results were also visualized in the scores and coefficient plots. MATLAB (software, version 7.1, MathWorks) produced the coefficient plots that were color‐coded with absolute value of correlation coefficients (*r*).

To get quantitative analysis data of metabolites, the level of metabolites was calculated by equating integrals of picked metabolite NMR signals (least overlapping ones) in relation to that of internal reference (TSP) with known concentration.

### 2.5 Sensory evaluation

Sensory analysis included 7 types of taste characteristics (sourness, sweetness, bitterness, umami, saltiness, after‐taste, and overall‐taste). The four meat samples were cut into thin slices (about 3 × 2 × 0.5 cm) and then randomly distributed to the trained panelists. The panelists (15 men and 15 women aged between 25 and 35) have undergone a selection and basic training to improve their ability before evaluates the taste of CSSB. They were trained to know the products and the methodology and were to focus on the evaluation of CSSB samples. The training lasted at least 2 weeks. Several types of CSSB were provided to the panelists during the training for providing a broad range of sensory variability for each taste characteristics and further stimulating the formation of descriptors. Each member needs to grade the taste qualities from 0 (not detectable) to 7 (strongly detectable) points and performed a descriptive analysis of Wanlong, Hengsheng, Pingyao, and Yueshengzhai and evaluated the attributes. Before the sensory evaluation, eating and smoking were not permitted for an hour. Drinking water was also provided to cleanse the mouth cavity between testing each meat sample.

## 3 RESULTS AND DISCUSSION

### 3.1 Sensory evaluation

**FIGURE 1:**
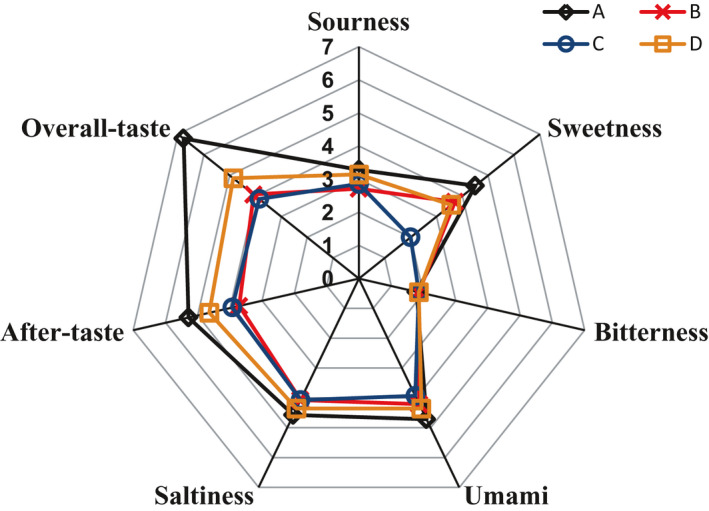
Sensory analysis results of 4 different Chinese sauce‐stewed beef. A = Wanlong, B = Hengsheng, C = Pingyao, D = Yueshengzhai

Figure 1 showed the taste profiles of CSSB from Wanlong (A), Hengsheng (B), Pingyao (C), and Yueshengzhai (D) samples. Sensory evaluation revealed that Wanlong owned the highest intensity of sourness, sweetness, umami, and saltiness except for bitterness, which lead to the highest score of after‐taste and overall‐taste. The score of sourness, umami, and saltiness in Wanlong and Yueshengzhai was higher than that of Pingyao and Hengsheng. Wanlong and Hengsheng had a stronger sweetness than Pingyao and Yueshengzhai. Compared with other taste, the score of bitterness is at a low level which indicated that bitterness is not felt strongly in all products. Pingyao owned the lowest score of sweetness, umami, and saltiness, which lead to the lowest intensity of overall‐taste. According to the score of overall‐taste, the taste differences did exist not only between northern and southern products but also between northern products and between southern products.

### 3.2 ^1^H NMR spectra of extracts from 4 styles of CSSB

**FIGURE 2:**
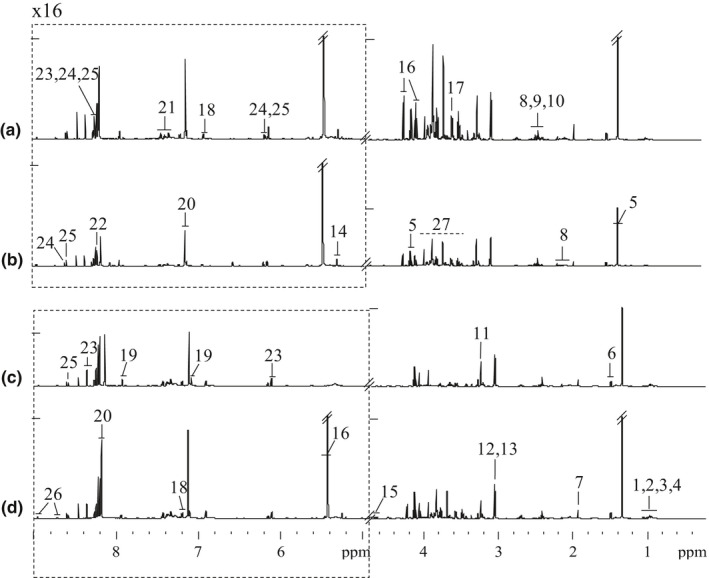
Four typical 600 MHz ^1^H nuclear magnetic resonance spectra of Chinese sauce‐stewed beef extracts from Wanlong (a), Hengsheng (b), Pingyao (c), Yueshengzhai (d). The dotted region was vertically expanded 16 times. Resonance assignments are given in Table 1. Keys: 1.isoleucine; 2. leucine; 3. valine; 4. 2,3‐butanediol; 5. lactate; 6. alanine; 7. acetate; 8. glutamate; 9. succinate; 10. pyroglutamate; 11. carnitine; 12. creatine; 13. creatinine; 14. α‐glucose; 15. β‐glucose; 16. sucrose; 17. glycine; 18. tyrosine; 19. histidine; 20. histamine; 21. phenylalanine; 22. hypoxanthine; 23. inosine; 24. AMP; 25. 5′‐IMP; 26. nicotinamide; 27. sugars and amino acids

Figure 2 showed representative ^1^H NMR spectra of CSSB extracts from Wanlong (A), Hengsheng (B), Pingyao (C), and Yueshengzhai (D). The resonances were assigned to detailed metabolites, and specifically identified with a series of 2D NMR experiments by both ^1^H and ^13^C data (Le, Colquhoun, Davis, Collins, & Verhoeyen, 2003). Twenty‐five metabolites were identified in the ^1^H NMR spectra; they were 10 amino acids (isoleucine, leucine, valine, alanine, glutamate, pyroglutamate, glycine, tyrosine, histidine, and phenylalanine), 2 sugars (glucose and sucrose), 1 alcohol (2,3‐butanediol), 4 organic acids (lactate, acetate, succinate, and creatine), 4 nucleotide metabolites (hypoxanthin, inosine, AMP, and 5′‐IMP), and 4 other metabolites (carnitine, histamine, nicotinamide, and creatinine). Metabolites such as isoleucine, leucine, glutamate, hypoxanthine, and inosine have been reported in fresh beef (Abraham, Dillwith, Mafi, Vanoverbeke, & Ramanathan, 2017; David, Juan Manuel, Rosa, Antonio, & María Isabel, 2015). However, metabolites like histamine, 2,3‐butanediol, and pyroglutamate were rarely detected before. As seen from Figure 2a–d, Wanlong (A) had higher total metabolites level than Hengsheng (B) and Yueshengzhai (D). The features of ^1^H NMR spectra of 4 styles of CSSB revealed significant difference.

### 3.3 Principal components analysis

**FIGURE 3:**
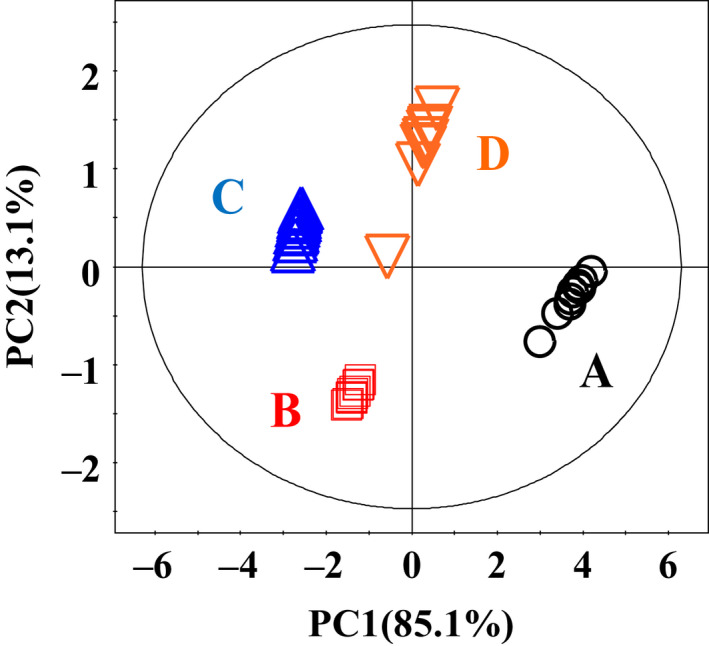
PCA scores plot for Chinese sauce‐stewed beef extracts from Wanlong (A), Hengsheng (B), Pingyao (C), and Yueshengzhai (D). PC1 and PC2 represent 85.1% and 18.1% of the total variance, respectively

Figure 3 showed the PCA results. PC1 and PC2 explained 85.1% and 13.1% of the total variables, respectively. PCA scores plot showed the obvious metabolites profile variation; 4 styles of CSSB were distributed in 4 different parts of the figure. PCA results clearly indicated the significant differences among them. Wanlong (A) owned the highest PC1 value among all products with highest score of overall‐taste showed in Figure 1. The PC1 value of Wanlong (A), Yueshengzhai (D), Hengsheng (B), and Pingyao (C) was declined gradually; the result was corresponding to the result of overall‐taste in Figure 1.

### 3.4 Metabolomics analysis and their contribution to taste

**FIGURE 4:**
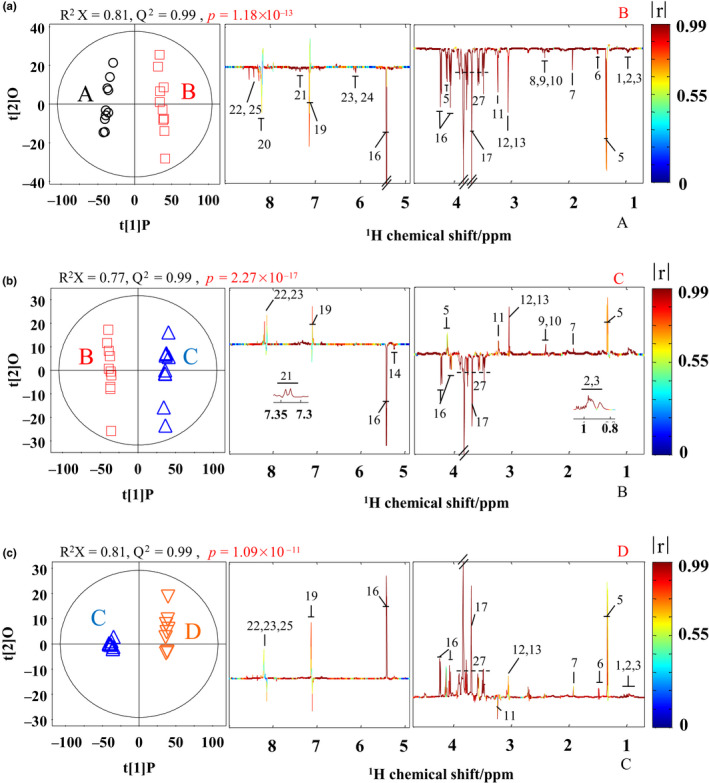
OPLS‐DA score plots (left) and corresponding color‐coded correlation coefficient loadings plots (right) originated from comparisons between spectra of Chinese sauce‐stewed beef extracts from Wanlong (A), Hengsheng (B), Pingyao (C), and Yueshengzhai (D). Metabolite identification keys to the numbers are displayed in Figure 2 and Table 1

To investigate the changes and discrimination of metabolites, the coupled contrasted OPLS‐DA scores plots and corresponding color‐coded correlation coefficient loadings plots were exhibited in Figure 4. The OPLS‐DA from Wanlong and Hengsheng (Figure 4a), Hengsheng and Pingyao (Figure 4b), Pingyao and Yueshengzhai (Figure 4c) spectral data were compared, respectively. In Figure 4a, compared with Hengsheng, Wanlong owned higher levels of isoleucine, leucine, valine, lactate, alanine, acetate, glutamate, succinate, pyroglutamate, carnitine, creatine, creatinine, sucrose, glycine, histidine, histamine, phenylalanine, hypoxanthine, inosine, AMP, and 5′‐IMP. In Figure 4b, compared with Pingyao, Hengsheng owned higher levels of α‐glucose, sucrose, and glycine, but lower level of leucine, valine, lactate, acetate, succinate, pyroglutamate, carnitine, creatine, creatinine, histidine, phenylalanine, hypoxanthine, and inosine. In Figure 4c, compared with Yueshengzhai, Pingyao owned higher levels of carnitine, but lower levels of isoleucine, leucine, valine, lactate, alanine, acetate, creatine, creatinine, sucrose, histidine, hypoxanthine, inosine, and 5′‐IMP. The results revealed the good matrix character and clear metabonomic difference between the contrastive groups.

The numerical values of R^2^X and Q^2^ on the OPLS‐DA scores plots showed rational quality of the contrastive groups. The color‐coded correlation coefficient loadings plots (Figure 4, right) represented significant difference. Hot‐colored sign explained more marked contribution, whereas the cold‐colored one represented less marked contribution. In present study, the differences in metabolites with correlation coefficients >0.602 were thought to be significant, which was equal to a significant level of discrimination against *p < *.05.

Table 1 (left part) showed the coefficients for identified metabolites to explain the significance of their contributions. Wanlong and Hengsheng samples showed obvious difference (*r > *.602) in all detected metabolic profiles not including histamine and hypoxanthine. It also showed obvious difference (*r > *.602) in all detected metabolic profiles excepted for β‐glucose, histamine, and hypoxanthine between Hengsheng and Pingyao samples. Among 4 styles of CSSB, the levels of most metabolites were the highest in Wanlong, especially the sucrose. On the contrary, Pingyao samples owned relatively lower levels of sucrose compared with others. The consequence in Table 1 (left part) was highly corresponded to the OPLS‐DA results.

**Table 1:** Coefficients from orthogonal projection to latent structure discriminant analysis and levels of metabolites of 4 styles of Chinese sauce‐stewed beef extracts

Metabolite	Coefficient^a^			Mean ± *SD* (mg/g)^c^			
	B/A^b^	C/B	D/C	A	B	C	D
Isoleucine	−0.99	−0.98	0.99	1.33 ± 0.05a	0.93 ± 0.04c	0.67 ± 0.01d	1.09 ± 0.07b
Leucine	−0.99	0.99	0.95	0.16 ± 0.00c	0.08 ± 0.00d	0.18 ± 0.00b	0.25 ± 0.02a
Valine	−0.99	0.99	0.98	0.09 ± 0.00b	0.04 ± 0.00d	0.08 ± 0.00c	0.14 ± 0.01a
Tyrosine	−0.99	0.99	0.96	0.08 ± 0.00b	0.04 ± 0.00c	0.08 ± 0.00b	0.15 ± 0.01a
Histidine	−0.81	0.77	0.85	1.31 ± 0.06b	0.67 ± 0.03d	1.04 ± 0.03c	1.83 ± 0.13a
Phenylalanine	−0.99	0.99	0.94	0.27 ± 0.01c	0.15 ± 0.00d	0.33 ± 0.00b	0.44 ± 0.03a
Alanine	−0.96	0.99	0.94	0.33 ± 0.01b	0.19 ± 0.00d	0.27 ± 0.00c	0.38 ± 0.03a
Glycine	−0.99	−0.98	0.99	/	/	/	/
Glutamate	−0.99	−0.92	0.96	1.33 ± 0.05a	0.62 ± 0.03c	0.56 ± 0.02d	0.76 ± 0.05b
Pyroglutamate	−0.99	0.99	0.97	1.38 ± 0.07b	0.76 ± 0.03d	1.06 ± 0.02c	1.52 ± 0.11a
α‐Glucose	0.64	−0.99	0.89	0.22 ± 0.03b	0.25 ± 0.02a	0.07 ± 0.01d	0.18 ± 0.02c
β‐Glucose	−0.64	─	0.91	0.39 ± 0.03b	0.32 ± 0.03c	0.34 ± 0.02c	0.54 ± 0.06a
Sucrose	−0.98	−0.99	0.99	16.52 ± 0.74a	5.96 ± 0.27c	0.13 ± 0.00d	6.72 ± 0.48b
Lactate	−0.96	0.66	0.76	4.12 ± 0.19b	2.25 ± 0.08d	2.98 ± 0.10c	4.33 ± 0.32a
Acetate	−0.99	0.98	0.97	0.18 ± 0.00a	0.10 ± 0.00d	0.11 ± 0.00c	0.17 ± 0.02b
Succinate	−0.99	0.99	0.89	0.28 ± 0.01b	0.18 ± 0.00d	0.26 ± 0.00c	0.30 ± 0.02a
Creatine	−0.91	0.95	0.94	1.17 ± 0.05a	0.89 ± 0.04d	0.94 ± 0.03c	1.08 ± 0.07b
Hypoxanthine	─	─	─	0.27 ± 0.01b	0.22 ± 0.01b	0.29 ± 0.03b	0.60 ± 0.35a
Inosine	−0.94	0.96	−0.99	0.30 ± 0.01a	0.16 ± 0.00d	0.20 ± 0.00c	0.21 ± 0.02b
AMP	−0.89	0.74	0.83	0.11 ± 0.00a	0.07 ± 0.00c	0.08 ± 0.00b	0.11 ± 0.00a
5′‐IMP	−0.95	−0.82	−0.96	0.14 ± 0.00a	0.11 ± 0.00b	0.06 ± 0.00d	0.08 ± 0.00c
Carnitine	−0.98	0.99	−0.92	3.76 ± 0.17a	2.36 ± 0.10c	2.68 ± 0.09b	2.45 ± 0.19c
Creatinine	−0.97	0.95	0.92	1.52 ± 0.07a	0.83 ± 0.04d	1.05 ± 0.03c	1.37 ± 0.09b
Histamine	─	─	─	/	/	/	/
Nicotinamide	−0.86	−0.95	0.96	0.04 ± 0.00b	0.04 ± 0.00b	0.03 ± 0.00c	0.06 ± 0.00a
2,3‐Butanediol	0.96	−0.72	−0.87	0.02 ± 0.00b	0.03 ± 0.00a	0.02 ± 0.00b	0.02 ± 0.00b

^a^ The coefficients from OPLS‐DA results, positive and negative signs indicate positive and negative correlation in the concentrations, respectively. The coefficient of 0.602 was used as the cutoff value for The significant difference evaluation (*p < *.05). “─” means the value of coefficient is lower than 0.602.

^b^ A, B, C, and D represent Chinese sauce‐stewed beef extracts obtained from Wanlong, Hengsheng, Pingyao, and Yueshengzhai, respectively.

^c^ The absolute concentration and standard deviation (mean ± *SD*, mg/g) were obtained from 10 parallel samples. “/” means the absolute concentration was not determined due to signal overlapping. “a–d” means significant difference among 4 styles of Chinese sauce‐stewed beef (*p < *.05).

An absolute quantification of identified metabolites was performed to get more information about the difference among 4 styles of CSSB. Individual metabolite level was showed in Table 1 (right part). The kinds of free amino acids (isoleucine, leucine, valine, alanine, glutamate, pyroglutamate, glycine, tyrosine, histidine, and phenylalanine) occupied more than half of identified metabolites. Mottram (1998) reported that FAAs were essential flavor precursors and contributed to the development of the taste of cooked meat. Most individual FAA had a decisive effect on the taste of dry‐cured hams (Careri et al., 1993). Zhang et al. (2018) had already demonstrated that FAAs played an important role in the taste of ham whether it is eastern ham or western ham. Among the 4 styles of CSSB, Yueshengzhai owned the highest levels of leucine, valine, tyrosine, histidine, phenylalanine, alanine, and pyroglutamate; Hengsheng owned the lowest levels of leucine, valine, histidine, phenylalanine, alanine and pyroglutamate. Rotolapukkila, Pihlajaviita, Kaimainen, and Hopia (2015) have found that the higher cooking temperature and longer time would contribute more to the hydrolysis of proteins into amino acids. However, it could be also originated from the different additive amount of soy sauce. Gao et al. (2011) reported that Chinese‐type soy sauce contains abundant FAAs such as aspartic acid, glutamate, alanine, glycine, serine, threonine, proline, lysine, arginine, histidine, isoleucine, leucine, methionine, phenylalanine, tryptophan, tyrosine, valine, and cysteine. Of these FAAs, leucine, isoleucine, glutamate, lysine, and arginine occupied more than 47% of total FAAs level.

Among identified FAAs, Wanlong keep the most abundant level of glutamate (1.33 ± 0.05 mg/g), which contributed to the pleasantly fresh taste (Watanabe et al., 2016). Fujimura et al. (1996) reported that the umami, sweet, and sour tastes of meat were enhanced by increasing glutamate level. Amino acids such as alanine have been shown to be closely associated with sweet flavor and palatable taste (Yang et al., 2017). In our study, Table 1 (right part) exhibited that Wanlong owned highest total levels of glutamate and alanine, while Hengsheng owned the lowest total levels of the two desirable FAAs among all products. This results suggesting that the flavor of Wanlong may be tasted for a higher sense of delicate flavor.

FAAs such as isoleucine, leucine, valine, tyrosine, histidine, and phenylalanine were associated with bitter taste (Kodani, Miyakawa, Komatsu, & Tanokura, 2017). The total levels of bitter FAAs were higher than umami and sweet FAAs. In Wanlong, Hengsheng and Yueshengzhai, isoleucine keeps a relatively higher level compared with other metabolites. However, bitterness was not perceived strongly in the 4 styles of CSSB though the bitter metabolite existed according to Figure 1. It might have been caused by bitter FAAs that partially suppressed by the presence of sodium chloride, sugars, and acids (Lioe, Apriyantono, Takara, Wada, & Yasuda, 2006). Breslin (1996) reported that phenylalanine and tyrosine can further improve the umami taste of a mixture solution of glutamic acid/sodium chloride. Nishimura, Ra Rhue, Okitani, and Kato (1988) also found that the intensity taste of chicken was more intense when the levels of FAAs aggrandized, especially valine, aspartic acid, leucine, isoleucine, tyrosine, and phenylalanine. Hence, we concluded that no specific amino acid characterized its unique taste, but all the FAAs combined to give the complex taste sensation.

Hypoxanthine, inosine, 5′‐IMP, and AMP were identified as nucleotide degradation products (Yang et al., 2017). AMP was degraded to 5′‐IMP; 5′‐IMP was degraded to inosine, and inosine to hypoxanthine; it was a gradual process of decomposition (Tikk et al., 2006). 5′‐IMP was the major nucleotide which was discovered in muscle and was thought to be one of the major precursors with umami taste in meat (Jayasena, Ahn, & Jo, 2013). Among 4 styles of CSSB, 5′‐IMP existed as the highest level in Wanlong and the lowest level in Hengsheng. The levels of inosine and AMP were significantly different (*p* < .05) among all products, but the difference of AMP level was not obvious between Wanlong (0.11 ± 0.00 mg/g) and Yueshengzhai (0.11 ± 0.00 mg/g). Hypoxanthine possesses bitter flavor characteristics (Bailey, 1983); the difference of hypoxanthine level is not obvious in Wanlong (0.27 ± 0.01 mg/g), Hengsheng (0.22 ± 0.01 mg/g), and Pingyao (0.29 ± 0.03 mg/g); Yueshengzhai owns the highest level of hypoxanthine. Arya and Parihar (1979) found that inosine and hypoxanthine level were increasing constantly in goat's and sheep's meat during cooking process and it could explain that the levels of inosine and hypoxanthine was higher than 5′‐IMP and AMP in the 4 styles of CSSB. It indicated clearly that nucleotide degradation products have a relatively lower level compared with other metabolites, but they are still supposed to be important composition in meat flavor formation and development (Tikk et al., 2006).

In regard to sugar, the level of sucrose is extremely high in Wanlong (16.52 ± 0.74 mg/g) and extremely low in Pingyao (0.13 ± 0.00 mg/g). Significant difference between the two styles of products reveals that the taste of China southern area tends to be sweet and of China northern area tends to be salty (Zeng et al., 2016). In Chinese stewed pork‐hock in soy sauce, a mass of sugar were added in the later time of stewing process with all sauce is absorbed (Yang et al., 2019). In our study, the extremely high level of sucrose in Wanlong may cause by adding large amount of sugar during stewing process. Wanlong and Hengsheng both belong to China southern product, but the level of sucrose in Wanlong was three times higher than that of Hengsheng. Pingyao and Yueshengzhai both belong to China northern product; the level of sucrose in the two products was significantly different. We speculate that the significant difference may cause by geographical location. The origin of Hengsheng is Anhui province that is at the north–south border in China; the taste of Hengsheng significantly influenced by north flavor. The origin of Yueshengzhai is Beijing which is the capital of China; the population of Beijing is composed of people with diverse taste from different regions which deeply affect the taste of north flavor.

Table 1 (right part) also showed the level of organic acids (lactate, acetate, succinate, and creatine). Hengsheng and Pingyao owned lower total levels of 4 organic acids than Wanglong and Yueshegzhai. Lactate was a mild acid and served the sour taste to cooked beef loins (Kang et al., 2013). The levels of lactate were 4.12 ± 0.19, 2.25 ± 0.08, 2.98 ± 0.10, and 4.33 ± 0.32 mg/g in Wanlong, Hengsheng, Pingyao, and Yueshengzhai. Among the 4 styles of CSSB, Yueshengzhai was the only halal product and owned the highest level of lactate. Due to the special slaughtering process, halal slaughter beef is different from common slaughtered beef. After halal slaughter, the speed of pH value decreased differently between halal beef and nonhalal beef. It could be the main reason which caused the highest level of lactate in Yueshengzhai. Creatine, a key compound, plays important role in muscle energy metabolism (Wyss & Kaddurahdaouk, 2000). Akma, Masashi, Makoto, Hideyuki, and Koretaro (2010) reported that creatine as a taste‐active component enhanced the characteristic flavor of thickness and mouthfulness. Among all products, Wanlong owns the highest level (1.17 ± 0.05 mg/g) of creatine, which 24%, 20%, and 7.7% higher than Hengsheng, Pingyao, and Yueshengzhai. Wanlong also owned the highest acetate level among 4 styles of CSSB.

Creatinine is an end‐product which transformed by creatine due to a nonenzymatic conversion through the elimination of water and the formation of a ring structure under heating condition (Massimiliana, Sandro, & Alessandro, 2008). The level of creatinine was significantly higher in Wanlong, Pingyao, and Yueshengzhai, and was higher than creatine in 4 styles of CSSB except for Hengsheng. Schlichtherle‐Cerny and Grosch (1998) reported that creatine and creatinine were both responsible for bitter taste. Azad, Ogasawara, Kurihara, and Takahashi (2013) also found that the two metabolites were important in the development of the characteristic taste and flavor of dried herring fillet. Carnitine was a ubiquitous constituent in mammalian plasma and tissues, mainly distributed among skeletal and cardiac muscles (Shimada et al., 2004). Rigault, Mazué, Bernard, Demarquoy, and Le (2008) reported that carnitine level exhibited no significant difference in raw and cooked beef, but in salmon, smoking process would cause two thirds of the carnitine lost. In our study, compared with other metabolites, carnitine has been discovered in relatively high level among 4 styles of CSSB. Wanlong owned a highest level of carnitine (*p* < .05) among them; the level of Pingyao, Yueshengzhai, and Hengsheng declined successively.

## 4 CONCLUSIONS

The results of taste‐active metabolites levels are coincident with the results of sensory evaluation. Wanlong and Yueshengzhai belong to strong‐taste type; Hengsheng belongs to middle type; Pingyao belongs to mild‐taste type. The higher isoleucine, glutamate, sucrose, lactate, creatine, and creatinine levels in Wanlong and Yueshengzhai lead to higher taste sensory score than Pingyao and Hengsheng. Compared with others, Pingyao owns the lowest metabolites such as isoleucine, histidine, and sucrose with palest taste. The metabolites levels of Hengsheng and Yueshengzhai are between Wanlong and Pingyao. For our study, ^1^H NMR spectroscopy coupled with multivariate data analysis was identified providing a profile of most metabolites to distinguish the taste difference of different Chinese sauce‐stewed beef.
